# Supplementary material for: Content-rich biological network constructed by mining PubMed abstracts
Source: BMC Bioinformatics. 2004 Oct 8;5:147. doi: 10.1186/1471-2105-5-147 (PMC528731; doi:10.1186/1471-2105-5-147)
Supplement: Additional File 2 — The original results of the above study (non-essential files are deleted to keep the file size under the limit set by BMC bioinformatics). [file 1471-2105-5-147-S2.bz2 › chilibotAdditionalFile2/dip10/693ID11994151E18018/html/TOLA_TOLB.html]

 


 **TOLA** and **TOLB** 
  
Found 36 abstracts in PubMed, retrieved 10.  
 

 What does Google say? 
 PDF only 
| .edu only 

---

**Interactive relationship** (e.g. stimulation, inhibition, etc)

**Neutral relationship**- Mutational analysis of the  **TolA**  C terminal domain of Escherichia coli and genetic evidence for an interaction between  **TolA**  and  **TolB** .  Ref: 12142433 J Bacteriol, 2002
- The  **TolA**   **TolB**  interaction was confirmed by cross linking experiments on purified proteins.  Ref: 11994151 Mol Microbiol, 2002
- These results demonstrate that the  **TolA**   **TolB**  interaction allows the formation of a trans envelope complex that brings the inner and outer membranes in close proximity.  Ref: 11994151 Mol Microbiol, 2002
- Moreover, we show that the interaction between  **TolA**  and  **TolB**  is required for the uptake of colicin A and for the membrane integrity.  Ref: 11994151 Mol Microbiol, 2002
- **TolB**  dimerizes and the periplasmic domain of  **TolA**  interacts with YbgF and  **TolB** .  Ref: 11994151 Mol Microbiol, 2002
- This region and the other regions responsible for the interaction with  **TolA**  and  **TolB**  have been mapped precisely within the colicin A N terminal domain and appear to be arranged linearly in the colicin sequence.  Ref: 11703658 Mol Microbiol, 2001
- The Tol Pal system function requires an interaction between the C terminal domain of  **TolA**  and the N terminal domain of  **TolB** .  Ref: 11994151 Mol Microbiol, 2002
- Our results indicate that the central domain of  **TolA**  TolAII is sufficient to interact with YbgF, that the C terminal domain of  **TolA**  TolAIII is sufficient to interact with  **TolB** ,.  Ref: 11994151 Mol Microbiol, 2002
- The isolation of suppressor mutants of  **tolA**  mutations in the  **tolB**  gene confirmed an interaction between TolAIII and the N terminal domain of  **TolB** .  Ref: 12142433 J Bacteriol, 2002

**Non-interactive relationship** (e.g. studied together, co-existance, homology, etc.)

- Analysis of polar mutations, reverse transcriptase PCR assays, and transcriptional fusion constructs with a promoterless lacZ gene revealed that the genes are arranged in two operons orf1 tolQ tolR  **tolA**   **tolB**  and oprL orf2.  Ref: 12486055 J Bacteriol, 2003
- In Pseudomonas putida, the seven genes, orf1, tolQ, tolR,  **tolA** ,  **tolB** , oprL, and orf2, which encode the proteins of this complex, are clustered in a 5.8 kb region of chromosomal DNA.  Ref: 12486055 J Bacteriol, 2003
- The Tol Pal system of Escherichia coli is composed of the YbgC, TolQ,  **TolA** , TolR,  **TolB** , Pal and YbgF proteins.  Ref: 11994151 Mol Microbiol, 2002
- The Tol Pal system of the Escherichia coli envelope is formed from the inner membrane TolQ, TolR and  **TolA**  proteins, the periplasmic  **TolB**  protein and the outer membrane Pal lipoprotein.  Ref: 11115123 Mol Microbiol, 2000
- **TolA** , TolQ, and TolR are inner membrane proteins,  **TolB**  is a periplasmic protein, and Pal, the peptidoglycan associated lipoprotein, is anchored to the outer membrane.  Ref: 11790745 J Bacteriol, 2002
- We consider a variety of different colicins, including A, B, D, E1, E3, Ia, M and N, that penetrate through the porins OmpF, FepA, BtuB, Cir and FhuA, to subsequently interact with a few targets in the periplasm, including  **TolA** ,  **TolB** , TolC and TonB.  Ref: 12423783 Biochimie< MedlineTA>Biochimie, 2002
- Colicin A interacts with  **TolA**  and  **TolB**  via distinct regions of its N terminal domain.  Ref: 11703658 Mol Microbiol, 2001
